# Complete mitochondrial genome of *Neuroctenus yunnanensis* Hsiao, 1964 (Hemiptera: Aradidae: Mezirinae)

**DOI:** 10.1080/23802359.2023.2288442

**Published:** 2023-12-18

**Authors:** Qian Wang, Xiaoshuan Bai, Hongge Qian

**Affiliations:** a College of Life Sciences and Technology, Inner Mongolia Normal University, Hohhot, PR China; b College of Bioscience and Biotechnology, Hunan Agricultural University, Changsha, PR China

**Keywords:** Genome sequence, mezirinae, taxonomic status

## Abstract

The complete *Neuroctenus yunnanensis* Hsiao, 1964, mitogenome was sequenced using an Illumina NovaSeq platform and submitted to GenBank (accession number: ON507991). The mitochondrial genome is a typical circular DNA molecule of 15,283 bp with 37 genes, including 22 tRNA genes, 13 protein-coding genes (PCGs), two rRNA genes, and a control region. Phylogenetic reconstruction validated the taxonomic status of *N. yunnanensis*, which was placed in the Mezirinae subfamily (Aradidae) and most closely related to *N. parus*. The mitochondrial genome data of *N. yunnanensis* provides a basis for genetic research.

## Introduction

*Neuroctenus yunnanensis* Hsiao, 1964 (Mezirinae, Aradidae) is primarily distributed in the autonomous regions of Yunnan and Tibet where they live in wet forests with decaying fallen wood and dead branch bark (Xiao 1964). To date, only three mitochondrial genomes of flat bugs have been sequenced from Mezirinae: *Brachyrhynchus hsiaoi* (Li et al. 2016), *B. triangulus* (Zhu et al. 2023), and *N. parus* (Hua et al. 2008). Here, we present the first complete mitochondrial genome of *N. yunnanensis* and analyze its phylogenetic relationships.

## Materials and methods

### Specimens

**Figure 1.**
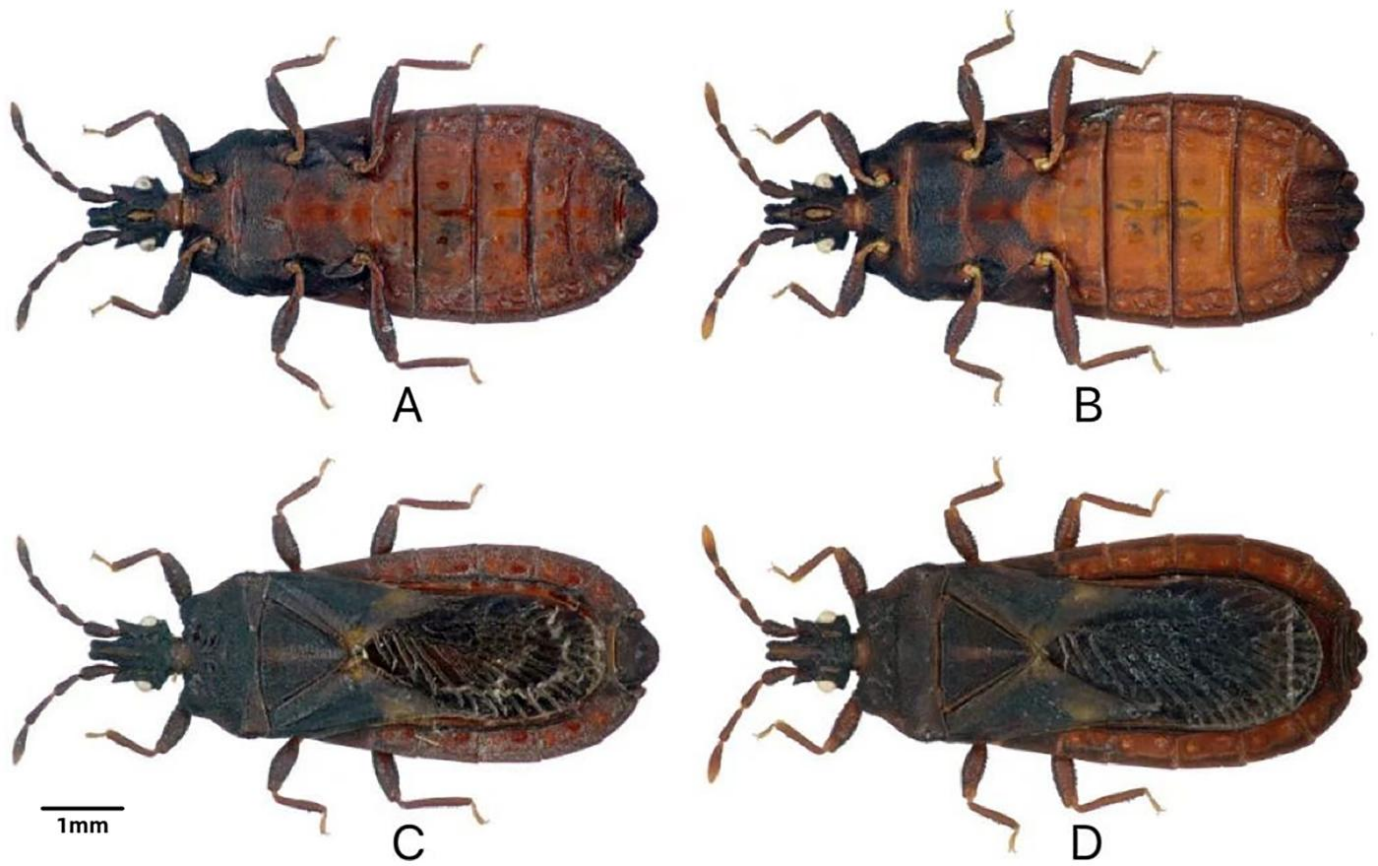
*Neuroctenus yunnanensis* Hsiao, 1964, reference image. A, B. male; C, D. female; A, C. dorsal view; B, D. ventral view (the photos were obtained by Xiaoshuan Bai at the Molecular lab, College of Life Science and Technology, Inner Mongolia Normal University, China).

Specimens of *N. yunnanensis* were collected in August 2019 from Yigongbai Village, Dexing Town, Motuo County, Linzhi City of the Tibet Autonomous Region, China (29.291832 N, 95.182762E, 335), and taxonomically identified by Professor Bai Xiaoshuan from Inner Mongolia Normal University (see Figure 1 for reference images). The specimen (voucher number: XLmt-1) was deposited at the Molecular Laboratory, College of Life Science and Technology, Inner Mongolia Normal University, China (http://bio.imnu.edu.cn/, Bai XS, baixs2007@aliyun.com). *N. yunnanensis* is an invertebrate that is neither endangered nor protected in China or other countries. Therefore, no ethical approval or other relevant permission was required for this study.

### DNA extraction, processing, and mitogenome annotation

Genomic DNA was extracted from the legs of *N. yunnanensis* using the 2 × CTAB method (Li et al. 2011). Complete mitochondrial genome sequencing and assembly were performed by Jishi Huiyuan Biotechnology using an Illumina NovaSeq platform and 2.93 GB of raw data were obtained. Mitochondrial genome assembly was performed using SPAdes v3.10.1 software (http://cab.spbu.ru/software/spades/), which is independent of reference genomes (Mapping Reads Number: 24,520, Average Coverage: 505.7878, Insert size: 282.86 ± 75.93). The assembly process comprised seven steps: (1) The SEED sequence was obtained by assembling mtDNA using SPAdes software. (2) Kmer iteratively extends the seed (if the result is a contig, proceed to 6). (3) Scaffolds were obtained by connecting the contig sequences using SSPACE 2.0. (4) Gapfiller software (v2.1.1) was used to fill the GAP of the scaffold sequences. (5) Assembly was repeated if there were gaps. (6) The sequences were aligned to the pseudogenome for genome correction. (7) The complete mitochondrial ring genome sequence was obtained after rearrangement according to the mitochondrial structure (Nurk et al. 2017). Mitos2 (http://mitos2.bioinf.uni-leipzig.de) was used to annotate the assembled genome sequence (E-value Exponent = 5; Maximum Overlap = 100; and ncRNA overlap = 100). Mitos2 annotation results were compared with those of closely related species, and the final annotation results were obtained after manual correction (Meng et al. 2019). The annotated mitogenome was deposited in GenBank (accession number: ON507991). Mitochondrial genomes were analyzed and counted using MEGA v.7.0 (Sudhir et al. 2018).

## Results

### Phylogenetic tree reconstruction

**Figure 2.**
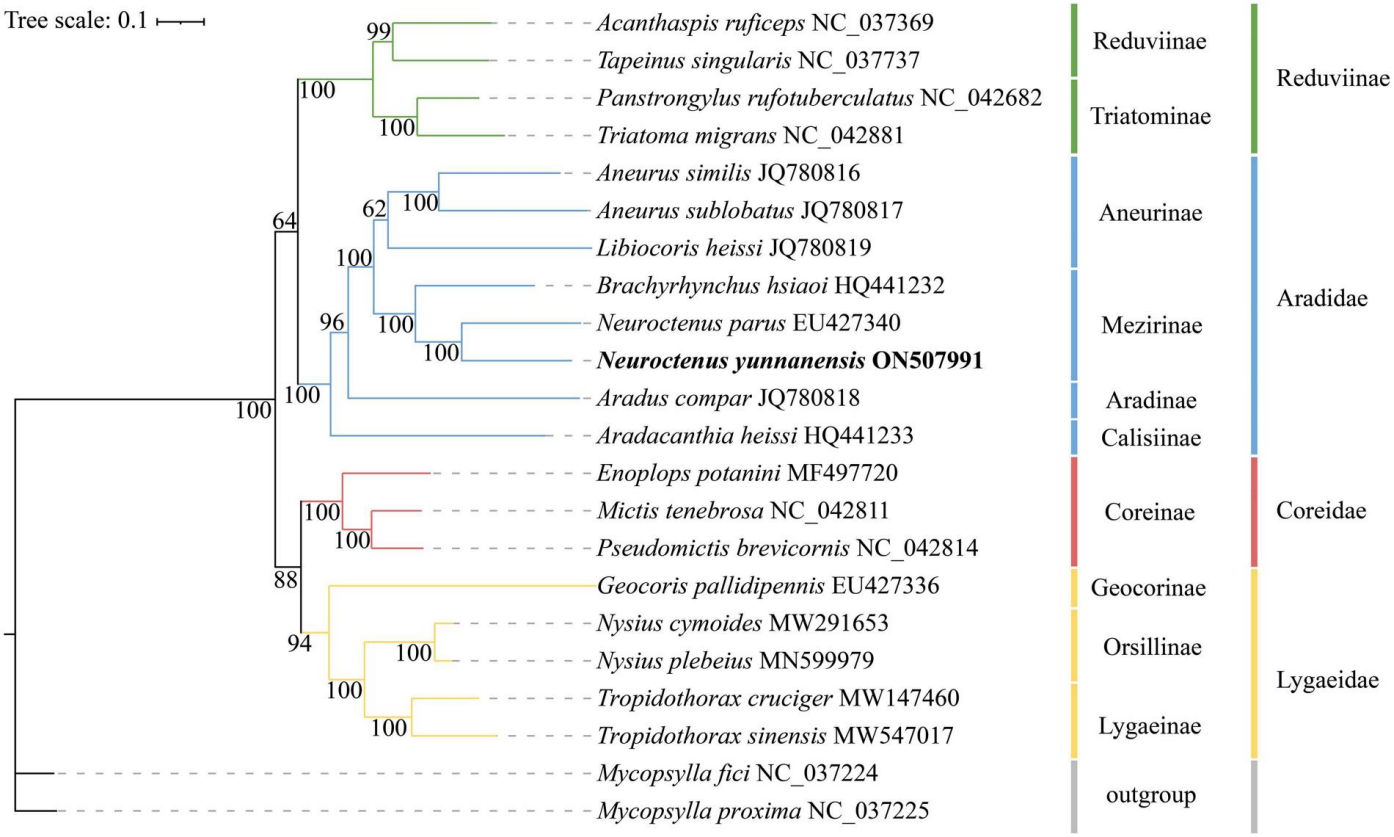
Phylogenetic tree obtained from maximum likelihood (ML) analysis based on PCGs of 22 species. The best-fit evolutionary model was GTR + F+I + G4.

To determine the phylogenetic position of *N. yunnanensis*, we downloaded the nucleotide sequences of 13 PCGs for 22 insect species from the NCBI database (Figure 2). (*N. yunnanensis*, as determined in the present study, had been added.) *Mycopsylla fici* and *M. proxima* were downloaded as outgroups, and 22 species were used to construct the phylogenetic trees. Maximum-likelihood (ML) (Nguyen et al. 2015) phylogenetic analysis based on the MAFFT (Kazutaka 2005) algorithm was performed using Phylosuite (Zhang et al. 2020).

### Mitochondrial genome characteristics

The mitochondrial genome of *N. yunnanensis* contains a typical circular DNA molecule of 15,283 bp. The A + T content was 69.34%, including 41.14% A, 28.20% T, 18.41% C, and 12.25% G. It encodes 22 tRNA genes, 13 PCGs, two rRNA genes, and a control region; 23 genes are encoded on the J-strand (major strand) and the remaining are oriented on the N-strand (minor strand), which was the same as for other Mezirinae (*B. hsiaoi* and *N. parus*). The A + T content of PCGs (69.04%), tRNA genes (70.44%), and rRNA genes (69.76%) are all higher than that of G + C. Among the 13 PCGs, the shortest was *ATP8* (156 bp) and the longest was *ND5* (1693 bp). Typical initiation codons ATN (where N represents A, G, or T) were found in most PCGs, except for the incomplete initial codons: *COI* (TT). All PCGs used TAA/TAG as the termination codon. All 22 tRNA genes in *N. yunnanensis* ranged from 60(*tRNA^Ala^*) to 71(*tRNA^Asp^*) bp. The lengths of *16S rRNA*, *12S rRNA*, and the control region were 1244 bp, 846 bp, and 768 bp, respectively.

## Discussion and conclusion

**Figure 3.**
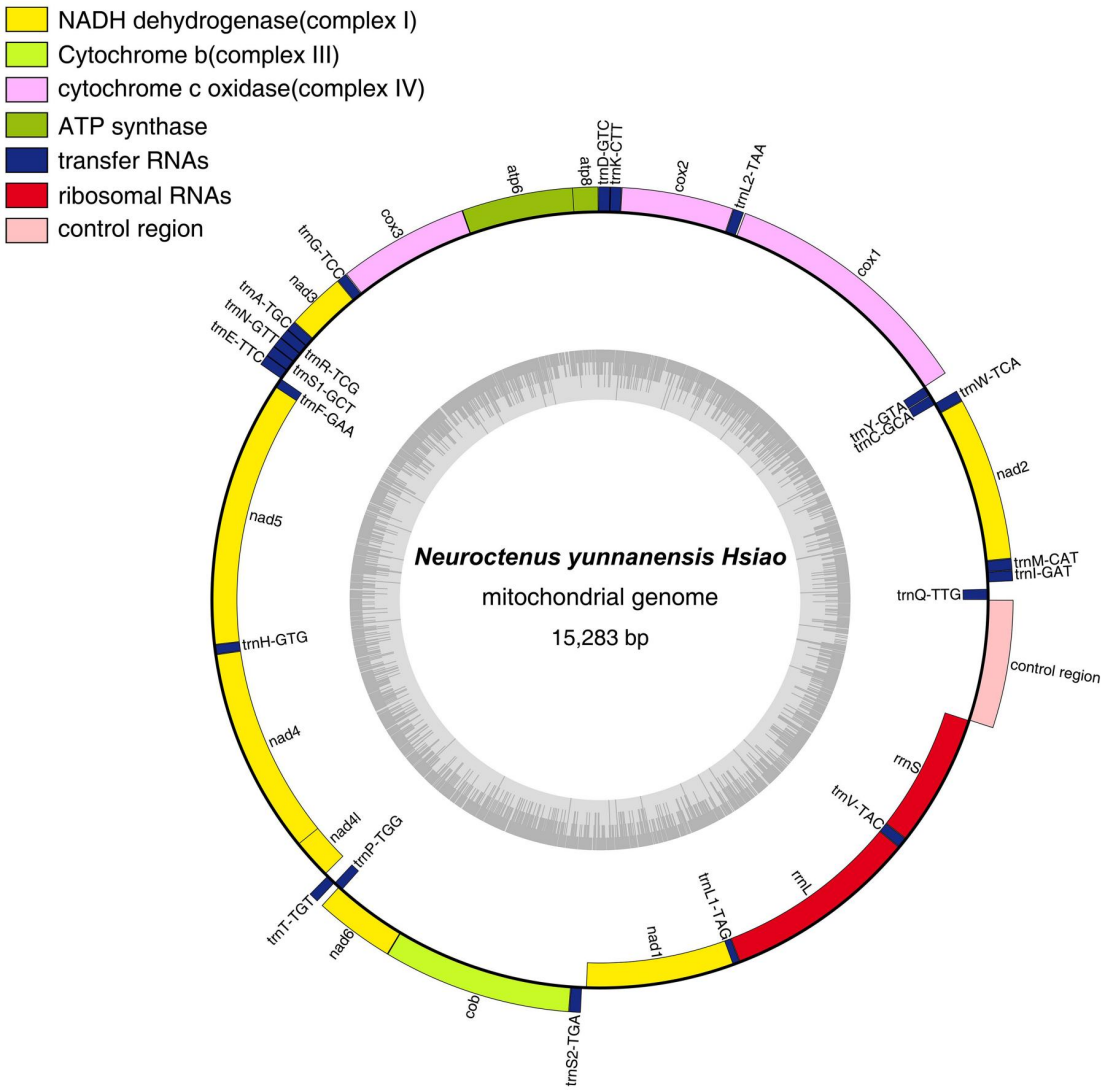
Gene map of the *N. yunnanensis* mitochondrial genome. Genes inside and outside the circle are transcribed clockwise and counterclockwise, respectively. The dark-gray inner circle corresponds to the GC content, and the light-gray circle corresponds to the at content.

We constructed a ML phylogenetic tree based on the 13 PCGs from 22 insect species (including two outgroups from Homotomidae). The phylogenetic tree topology structure was (Lygaeidae + Coreidae+(Reduviidae + Aradidae)) (Figure 3). The analysis confirmed that *N. yunnanensis* be assigned to the subfamily Mezirinae (Aradidae) and is most closely related to *N. parus*. Thus, the phylogenetic relationships of *N. yunnanensis* were consistent with traditional taxonomy.

In conclusion, the mitochondrial genome of *N. yunnanensis* is a typical circular DNA molecule of 15,283 bp with 37 genes, including 22 tRNA genes, 13 PCGs, 2 rRNA genes, and a control region. Phylogenetic reconstruction validated the taxonomic status of *N. yunnanensis* in the subfamily Mezirinae (Aradidae) and close relationship to *N. parus*. The mitochondrial genome data of *N. yunnanensis* provide the basis for genetic studies in the fields of insect taxonomy and resource conservation.

## Supplementary Material

Supplemental Material

Supplemental Material

Supplemental Material

## Data Availability

The genome sequence data that support the findings of this study are openly available in GenBank (NCBI, accession number: ON507991) (https://www.ncbi.nlm.nih.gov/). The associated Bio-Project, SRA, and Bio-Sample numbers were PRJNA768533, SRR16204191, and SAMN22047730, respectively.
