# Supplementary figures and images for: Complete mitochondrial genome of Neuroctenus yunnanensis Hsiao, 1964 (Hemiptera: Aradidae: Mezirinae)
Source: Mitochondrial DNA B Resour. 2023 Dec 18;8(12):1373–6. doi: 10.1080/23802359.2023.2288442 (PMC10732186; doi:10.1080/23802359.2023.2288442)

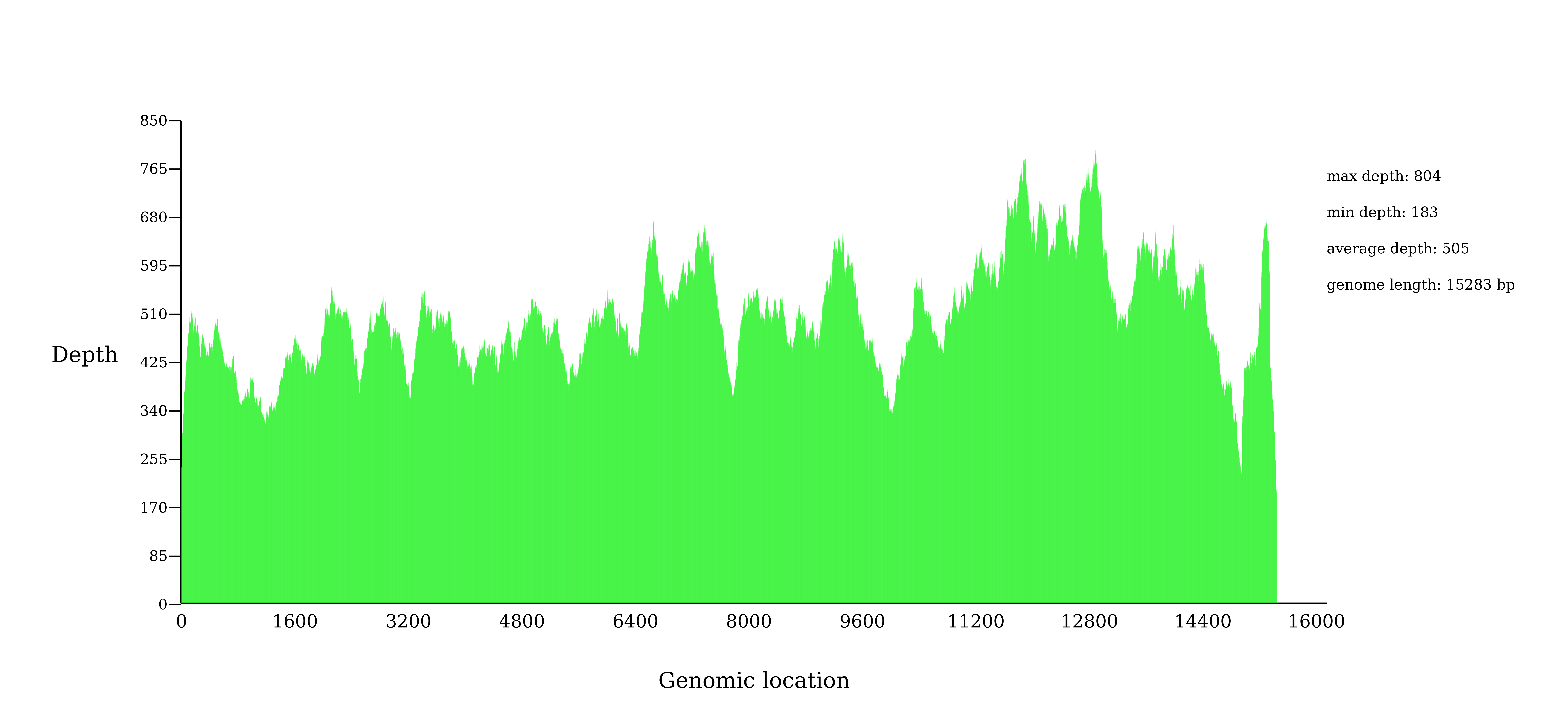

Supplement: Supplemental Material [file TMDN_A_2288442_SM4616.png]

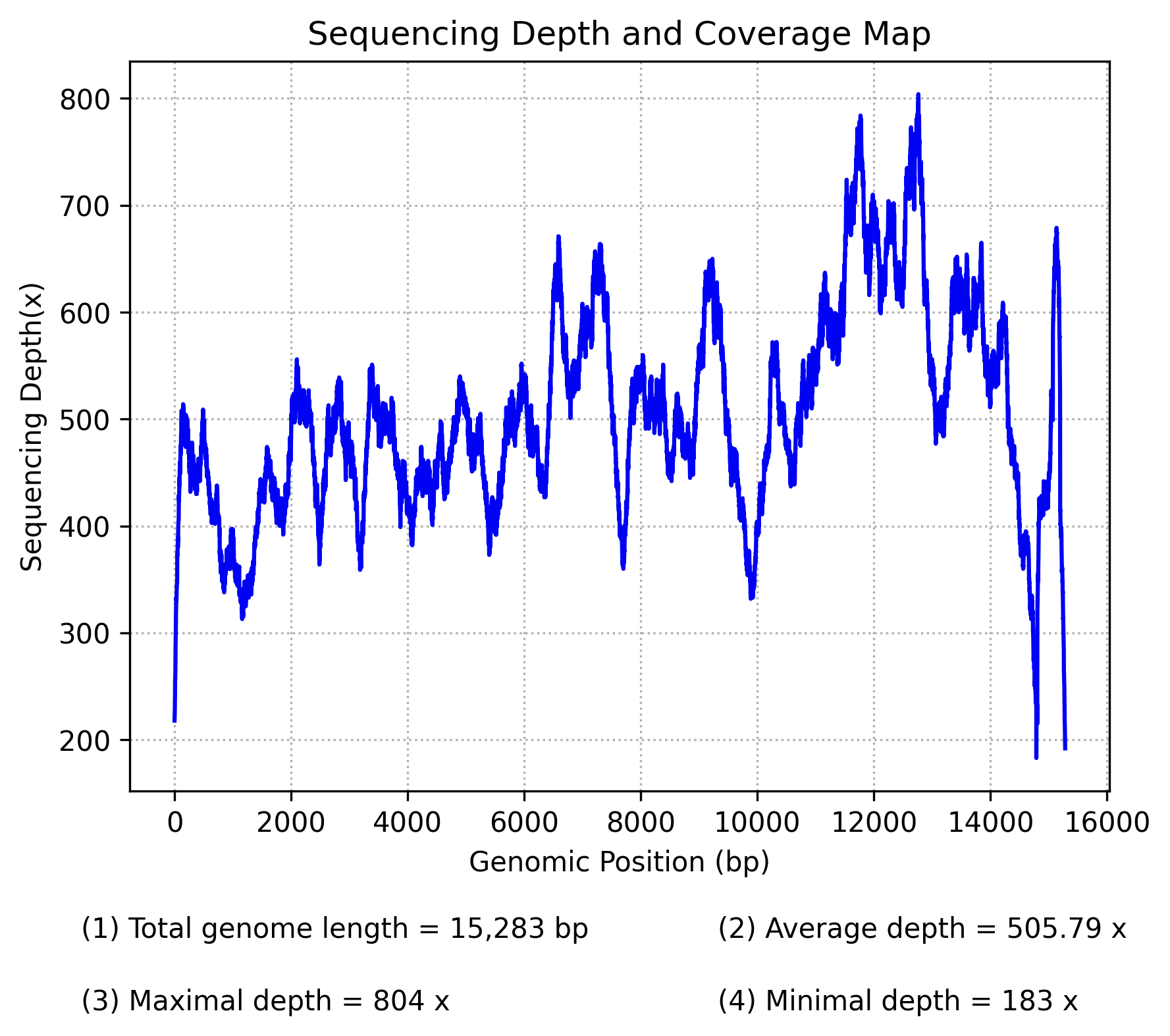

Supplement: Supplemental Material [file TMDN_A_2288442_SM4609.png]
