# Supplementary material for: Complete mitochondrial genome of Neuroctenus yunnanensis Hsiao, 1964 (Hemiptera: Aradidae: Mezirinae)
Source: Mitochondrial DNA B Resour. 2023 Dec 18;8(12):1373–6. doi: 10.1080/23802359.2023.2288442 (PMC10732186; doi:10.1080/23802359.2023.2288442)
Supplement: Supplemental Material [file TMDN_A_2288442_SM4608.pdf]

CERTIFICATE OF ENGLISH EDITING

This document certifies that the paper listed below has been edited to ensure that the language is clear and free of errors. The logical presentation of ideas and the structure of the paper were also checked during the editing process. The edit was performed by professional editors at Editage, a division of Cactus Communications, in cooperation with Taylor & Francis Group. The intent of the author's message was not altered in any way during the editing process. The quality of the edit has been guaranteed, with the assumption that our suggested changes have been accepted and have not been further altered without the knowledge of our editors.

Title

Complete mitochondrial genome of Neuroctenus yunnanensis Hsiao, 1964

Authors

QianWang

Order No.

IHXSC_1

EDITINGSERVICES

Supporting Taylor & Francis authors

Signature

Vikas Narang

Vikas Narang,
Chief Operating Officer,
Editage

Date of Issue
August 24, 2023

INFORMATION SECURITY MANAGEMENT SYSTEM DNV·GL ISO/IEC 27001

editage
